# Lipofection of Single Guide RNA Targeting *MMP8* Decreases Proliferation and Migration in Lung Adenocarcinoma Cells

**DOI:** 10.3390/medicina57070710

**Published:** 2021-07-14

**Authors:** Oscar David Hernandez Maradiaga, Pooi Ling Mok, Gothai Sivapragasam, Antony V. Samrot, Mohammed Safwan Ali Khan, Aisha Farhana, Badr Alzahrani, Jiabei Tong, Thilakavathy Karuppiah, Narcisse M. S. Joseph, Suresh Kumar Subbiah

**Affiliations:** 1 Department of Medical Microbiology and Parasitology, Universiti Putra Malaysia, Serdang 43400, Malaysia; gachetmd@gmail.com (O.D.H.M.); hanfabin1@gmail.com (J.T.); narcissems@upm.edu.my (N.M.S.J.); 2 Department of Clinical Laboratory Sciences, College of Applied Medical Sciences, Jouf University, Sakaka P.O. Box 2014, Saudi Arabia; afarhana@ju.edu.sa (A.F.); baalzahrani@ju.edu.sa (B.A.); 3 Department of Biomedical Science, Faculty of Medicine and Health Sciences, Universiti Putra Malaysia, Serdang 43400, Malaysia; thilathy@upm.edu.my; 4 Genetics and Regenerative Medicine Research Group, Universiti Putra Malaysia, UPM Serdang 43400, Malaysia; 5 Institute of Bioscience, Universiti Putra Malaysia, Serdang 43400, Malaysia; gothai_86@yahoo.com; 6 School of Bioscience, Faculty of Medicine, Bioscience and Nursing, MAHSA University, Jenjarom 42610, Malaysia; antonysamrot@gmail.com; 7 Department of Biomedical Sciences, School of Medicine, Nazarbayev University, Nur-Sultan 010000, Kazakhstan; safwanaucp@gmail.com; 8 Department of Pharmacology, Hamidiye International Faculty of Medicine, University of Health Sciences, Mekteb-I, Tibbiye-I Sahane (Hamidiye) Complex Selimiye Mahallesi, Tibbiye Caddesi #38, Istanbul 34668, Turkey; 9 Department of Biotechnology, Bharath Institute of Higher Education and Research, 173, Agaram Main Rd, Selaiyur, Chennai 600073, India

**Keywords:** CRISPR-Cas9, single guide RNA, lipofection, lung cancer, epithelial to mesenchymal transition (EMT), matrix metalloproteinase 8 (*MMP8*)

## Abstract

*Background and Objectives*: Matrix metalloproteinases (MMP) have been implicated as major determinants of tumour growth and metastasis, which are considered two of the main hallmarks of cancer. The interaction of *MMP8* and other signalling molecules within and adjacent tumoral tissues, including immune cells, are rather elusive, particularly of adenocarcinoma cell type. In this study, we aimed to investigate the role of *MMP8* in non-small cell lung cancer proliferation and invasiveness potential. *Materials and Methods*: We individually lipofected with two different single guide RNA (sgRNAs) that specifically targeted on *MMP8*, with CRISPR-Cas 9 protein into the cells. *Results*: Our results clearly indicated that the lipofection of these complexes could lead to reduced ability of A549 cells to survive and proliferate to form colonies. In addition, when compared to non-transfected cells, the experimental cell groups receiving sgRNAs demonstrated relatively decreased migration rate, hence, wider wound gaps in scratch assay. The quantitative real time-polymerase chain reaction (qRT-PCR) demonstrated significant reduction in the *MAP-K*, *survivin* and *PI3-K* gene expression. *MMP8* might have protective roles over tumour growth and spread in our body. *Conclusions*: The delivery of sgRNAs targeting on the *MMP8* gene could induce tumour cell death and arrest cell migratory activity.

## 1. Introduction

Adenocarcinoma represents the most common histology found in patients diagnosed with non-small cell lung carcinoma (NSCLC), representing around 40% frequency in the group of lung malignancies. This malignancy is usually located peripherally in the lung parenchyma [1,2], and can metastasize predominantly to the bone and respiratory system [3,4,5]. For distant metastases, the evidence-based outcome showed poor prognosis with only five per cent of patients may survive within five years [6,7]. Therefore, it is crucial to delve deeper into the signalling pathways, particularly the role of matrix metalloproteinases in the cascades that result in affected cell adhesion to acquire the ability to migrate and invade, i.e., epithelial to mesenchymal transition (EMT), new vessel formation and tumour spread [8].

Matrix metalloproteinase 8 (*MMP8*) is largely secreted by neutrophils and malignant cells [9]. To date, the function of *MMP8* in cancer progression has been confusing as various types of cancers demonstrated different prognosis outcome, despite presenting a similarly high level of *MMP8* expression. For example, in the skin, breast and oral tongue cancers, the presence of *MMP8* seems to confer protective anti-tumoral activities to patients by limiting cancer cell growth, invasion and spread. On the other hand, a high level of *MMP8* reduces the recovery rate and worsens the life expectancy of gastric and liver cancer patients [10].

Meanwhile, a previous study has shown that *MMP8* protein expression was significantly high in lung tissues samples from patients diagnosed with squamous cell carcinoma, as well as recurrent cases. However, *MMP8* protein level did not change in adenocarcinoma histology type when compared with normal tissue regions in the same patients [11]. In another study, treatment with hepatocyte growth factor could upregulate the expression of *MMP8* in lung adenocarcinoma cells, which in turn, enhanced the intercellular adhesion, thereby alleviating the migration activity of cells [12]. Similarly, previous studies indicate the presence of *MMP8* variants caused by single nucleotide polymorphism, such as rs2155052, rs35866072 and rs34009635 in populations, associated with reduced or no effect on the risks of developing lung cancer [13].

The migration and invasion potential in lung adenocarcinoma cells is promoted by a few MMP proteins, for example *MMP1* has been associated to migration and invasion in lung adenocarcinoma resistant to epidermal growth factor receptor tyrosine kinase inhibitors (*EGFR*-TKI) [14]. Similarly, the expression of *MMP2* and *MMP9* is high in lung adenocarcinoma and both proteins promote invasion and metastasis [15,16]. We have previously reported that targeting of *MMP20* gene in lung adenocarcinoma cells could stop the growth and reduce motility in the culture [17]. Nevertheless, there are limited data regarding the direct effect of *MMP8* molecule on the cellular behaviour of lung adenocarcinoma cells. This study examines the in vitro changes in the migration and proliferative potentials of A549 adenocarcinoma cells, along with its apoptotic activity, when the single guide RNA (sgRNA) targeting on *MMP8* was delivered by lipofection together with Cas9 protein. Much of the literature has shown that CRISPR-Cas9 gene editing method could efficiently and precisely cut on specific nucleotide sites, thereby inactivating the gene [18,19]. Our results provided evidence that treatment of cancer cells with lipofection complexes containing the sgRNA and Cas9 protein could lead to reduction of cell migratory and proliferative activity of adenocarcinoma lung cancer, besides promoting apoptosis. 

## 2. Materials and Methods

### 2.1. Cell Culture

The A549 (human lung adenocarcinoma) cell line was acquired as a gift from the UPM-MAKNA Cancer Research Laboratory Institute of Bioscience, Universiti Putra Malaysia (Serdang, Selangor). The cells were seeded at an initial density of 0.7 × 10^6^ in a 25-cm^2^ plastic flask containing 5 mL of Roswell Park Memorial Institute 1640 Medium (RPMI 1640; Nacalai Tesque Inc.; Kyoto, Japan) supplemented with 10% heat-inactivated Foetal Bovine Serum (FBS) (Gibco; Waltham, MA, USA) and 1% penicillin/streptomycin (Nacalai Tesque Inc.). The flask was then placed into a 37 °C humidified incubator supplied with 5% carbon dioxide (CO_2_) and the flask was routinely monitored. The cells were subcultured upon reaching 80% cell confluence with standard trypsinization method using 2.5 g/L trypsin–ethylenediaminetetraacetic acid (EDTA) (Nacalai Tesque Inc.). The trypsinized cells were washed with 1 X Phosphate Buffered Saline solution (PBS; Gibco) and then centrifuged at 1200× *g* for 5 min. Following centrifugation, the cell pellet was re-seeded into new sterile culture flasks at the same initial cell density.

### 2.2. Transfection of gRNA and Cas9 Protein Complexes into A549 Cell Line

For lipofection, two gRNAs with pre-designed sequences were purchased from Integrated DNA Technologies (Singapore). The Hs.Cas9.MMP8.1.AA (5′- CAAGUUAGUGCGUUCCCACU-3′) targets on the position of 102718433 on the negative strand while Hs.Cas9.MMP8.1.AB (5′-CUCCCUCAAGAUGACAUCGA-3′) targets on the position of 102722623 on the positive strand of *MMP8* gene.

Lipofection of these individual gRNAs into A549 cells was performed on a 96-well plate. One day before transfection, the A549 cells were seeded at an initial density of 1 × 10^4^ cells per well in a 96-well plate and placed into a CO_2_ incubator to achieve 70% confluence. First, 50 ng of gRNA was added to 5 µL of Opti-MEM medium (Gibco) containing 250 ng of TrueCut™ Cas9 protein v2 (Invitrogen) and 0.5 µL of Lipofectamine Cas9 Plus™ Reagent (Invitrogen) in a microcentrifuge tube. Meanwhile, in a separate tube, 5 µL of Opti-MEM medium (Opti-MEM™ I, New York, NY, USA) was mixed with 0.3 µL of Lipofectamine CRISPRMAX Cas9 Transfection Reagent (Invitrogen) and incubated for 1 min at ambient temperature (25 °C). The solutions from both tubes were then mixed together by gentle pipetting and allowed to incubate at ambient temperature for 15 min. Following that, 10 µL of the mixture solution was added to the A549 cells in each well. The 96-well plate was then placed into a CO_2_ incubator at 37 °C for 48 h. After incubation, the culture medium was gently removed and the cells were rinsed with 50 μL of PBS. The cells were trypsinized for further downstream experiment to determine transfection cytotoxicity and other cell behaviour affected by the gRNA. For studying the effect of lipofection of sgRNA on *MMP8*, non-transfected control cells were prepared by replacing sgRNA with deionized water only.

### 2.3. Colony-Forming Assay

To determine the colony forming seeding of the transfected A549 cells was performed in a 6-well plate at a density of 100 cells per well in normal culture medium then further grown for another 14 days. Then, the culture medium was gently removed and briefly washed with 1 X PBS solution. The cells were then fixated with 500 μL of 25% (*v/v*) acetic acid in methanol for 10 min at room temperature. The fixative was then removed and washed 3 times with 1 X PBS solution before staining with 0.05% (*w/v*) crystal violet stain solution at room temperature for 30 min. After that, the stain was gently removed and the cells were washed again with PBS. Stained colonies were then counted and photographed.

### 2.4. Cell Migration Assay

The scratch assay on A549 cells was performed, as previously described, in order to study the cell migration activity [20]. In brief, we performed seeding of the transfected and non-transfected cells into a 12-well plate at a density of 1 × 10^6^ cells per well, then the plate was placed into a CO_2_ incubator at 37 °C for 24 h until confluency in the monolayer was achieved. A scratch wound was created on the monolayer cell by using a sterile 200 µL plastic micropipette tip. The wound width was observed at 40 × total magnification. The wounded cell monolayer was incubated at 37 °C for 48 h in Opti-MEM™ I Reduced Serum Medium containing the treatment previously described. Images of the wound area were observed at 0 and 48 h using an inverted phase-contrast microscope. The assay was performed in triplicate, the areas in the scratch wound were captured and the wound width was determined and analysed by using ImageJ software. The formula below was used to quantitate the rate of cell migration and the value was reported in the unit of nm per hour (nm/h):

RM = (Wi − Wf)/t

RM = Rate of cell migration (nm/h); Wi = Initial wound width (nm); Wf = Final wound width (nm); t = Duration of migration (hour)

### 2.5. Cell Viability Assay

Apoptotic changes after lipofection were assessed by Acridine Orange/Propidium Iodide stain (AO/PI) double stain. In brief, 10 μL of the transfected cellular pellet was added with an equal volume of staining solution containing Acridine Orange (10 μg/mL) and Propidium Iodide (10 μg/mL) into the cell. Then, the solution was placed on the surface of a clean glass slide and covered using a coverslip. The slide was then observed under ultraviolet light in the fluorescence microscope within 30 min.

Meanwhile, the cytotoxic effects of each component used in the delivery of ribonucleoprotein complexes into the cells were determined by using 3-[4, 5-methylthiazol-2-yl]-2, 5-diphenyl-tetrazolium bromide (MTT) (Ruibio: Zhejiang, China) assay according to the protocol recommended by the manufacturer. 

The A549 cells were seeded into 96-well plates at a density of 1 × 10^4^ cells per well followed by incubation in 5% humidified CO_2_ incubator at 37 °C for 24 h. Next, the medium was gently replaced with reduced serum medium (Opti-MEM™ I, New York, NY, USA) and the individual tested compounds (Hs.Cas9.MMP8.1.AA sgRNA, Hs.Cas9.MMP8.1.AB sgRNA, Cas 9 protein, Cas 9 plus reagent and Lipofectamine CRISPRMAX) were added to each well of the plate. After incubation for 48 h, the medium was gently removed then 40 µL of MTT reagent was pipetted carefully in each well, then the plates were incubated at 37 °C for 4 h. Next, the reagent was gently aspirated and 100 µL of dimethyl sulfoxide (DMSO) was added to each well. Then the plates were placed into a CO_2_ incubator at 37 °C for 10 min to allow the development of colour. The absorbance of this solution was then quantified using an ELISA plate reader and the wavelength was set at 570 nm. The reading was also normalized by the absorbance value obtained from a reference wavelength (650 nm). The number of viable cells, following the addition of individual compound, was compared with wild type cells. Three independent experiments were performed in triplicate. 

### 2.6. RNA Isolation and Relative mRNA Expression

Transfected and non-transfected A549 cells were subjected to total RNA extraction into 50 μL elutes using the RNeasy^®^ Mini kit (QIAGEN, Valencia, CA, USA). Then, to synthesize cDNA from total RNA the SuperScript^®^ IV reverse transcriptase (Invitrogen, Carlsbad, CA, USA) kit was used as recommended by the manufacturer. Quantitative RT-PCR using THUNDERBIRD^®^ SYBR^®^ qPCR Mix (Toyobo, Osaka, Japan) on a Light Cycler^®^ 480 Real-Time PCR System (Roche Molecular Systems, California, USA) was performed to determine the expression levels of *MAP kinase (MAP-K), survivin* and *Phosphoinositide 3-kinase (PI3-K)* and the results were normalised using *β-Actin* as an internal control. Relative mRNA expression was calculated by the 2−ΔΔCt method. The sequences of the primers used for real time PCR are as follows: *β-Actin* F- TGGCACCCAGCAC AATGAA; R- CTAAGTCATAGTCC GCCTAGAAGC, *MAP-K* F-CAGTTCTTGACCCCTGGTCC; R-GTACATACTGCCGCAGGTCA, *survivin* F-CCAGACGATGACCCATGGAC; R-TGAAGAACTCTGCCACCGTC, *PI3-K* F-GGTGAAGCTCGTGTGTGGA; R-GAAGACAGGGCTCCACTTCC. 

### 2.7. Statistical Analysis 

For the one-way Analysis of Variance (ANOVA) and the post hoc Tukey’s multiple comparison test, Prism 8.0 (GraphPad Software, Inc., San Diego, CA, USA) software was used, *p* values less than 0.05 were considered to be statistically significant. The data were expressed as mean values ± Standard Error of the Mean (S.E.M).

## 3. Results

### 3.1. Treated Cells Showed Reduced Cellular Adhesion and Migration Compared to Non-Transfected Cells

**Figure 1:**
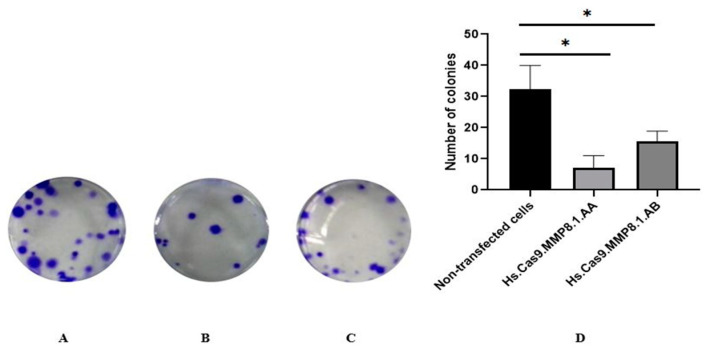
Colony formation decrease of A549 cells on treatment with Hs.Cas9.MMP8.1.AA and Hs.Cas9.MMP8.1.AB gRNAs for 14 d. (**A**) Non-transfected cells, (**B**) Hs.Cas9.MMP8.1.AA, (**C**) Hs.Cas9.MMP8.1.AB. (**D**) Number of colonies. The experiment was performed in two treatment groups and was compared with the non-transfected cells (* *p* < 0.05).

To determine whether the delivery of Cas9 protein and individual sgRNA into A459 cells could affect its cellular adhesion, therefore we performed colony formation assay. The colony formation assay could indirectly reflect the proliferative and adhesion ability of the cells. A colony consists of at least 50 cells. As represented by Figure 1, a remarkable decrease of colony formation in A549 cells was observed after 14 days following treatment with Hs. Cas9.MMP8.1. AA (Figure 1B) and Hs.Cas9.MMP8.1.AB gRNAs (Figure 1C), respectively. Meanwhile, in the non-transfected group, the cells showed few forming colonies and appeared dense in cultured (Figure 1A). Both treatment groups exhibited a significant number of colonies when compared to non-transfected group (7 and 15 vs. 32; *p* < 0.05) (Figure 1D). 

### 3.2. Treatment Decreased the Migration Potential Compared to Non-Transfected Cells

**Figure 2:**
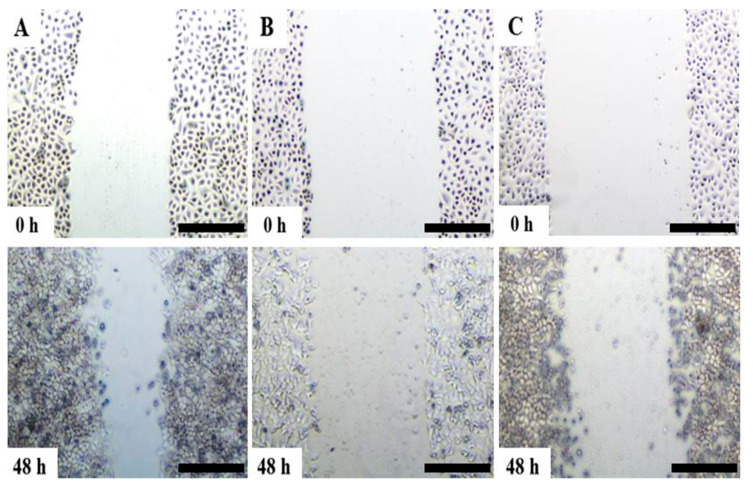
Migration inhibitory effects of Hs.Cas9.MMP8.1.AA (**B**) and Hs.Cas9.MMP8.1.AB (**C**) on A549 cells. The treatment on A549 cell line was observed for cell migration rate by using scratch assay. The scratch on A549 cell monolayer was created with 200 µL pipette tips and the cells were then viewed under microscope at 0 h prior to treatment. After 48 h, the distance of cell migration was analysed and measured. The treatment groups were compared with the control non-transfected cells (**A**). Scale 500 µm. (*n* = 3).

**Figure 3:**
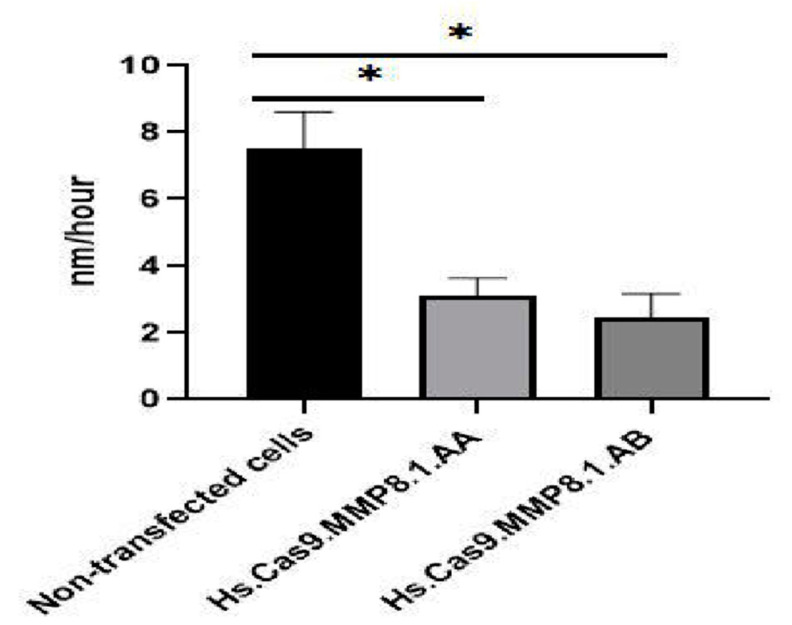
Effects of Hs.Cas9.MMP8.1.AA and Hs.Cas9.MMP8.1.AB gRNAs on rate of cell migration. The migration rate of A549 cells was followed up at 0 and 48 h. After treatment, the cell migration was analysed with ImageJ software and the rate was represented in nm/h from the initial gap between the two edges of each wound. The data were expressed as mean values ± Standard Error of the Mean (S.E.M) (*n* = 3) (* *p* < 0.05).

To determine whether the delivery of Cas9 protein and individual sgRNA into A459 cells could affect cellular migration, scratch assay was performed. Non-transfected and transfected monolayer cells were scratched with a pipette tip, and the ability of A549 cells to migrate and close the wound was observed. The results were indicated in Figure 2 and Figure 3. After 48 h following scratches, it was observed that in comparison to non-transfected control cells (Figure 2A), Hs.Cas9.MMP8.1.AA and Hs.Cas9.MMP8.1.AB gRNAs showed relatively wider wound gaps (Figure 2B,C). When measured with ImageJ, both treatment groups showed significant reduced migratory activities when compared to the non-transfected group (3.11 and 2.45 vs. 7.51 nm/h; *p* < 0.05) (Figure 3).

### 3.3. Treatment Triggered Apoptosis in Adenocarcinoma Lung Cells Compared to Non-Transfected Cells

**Figure 4:**
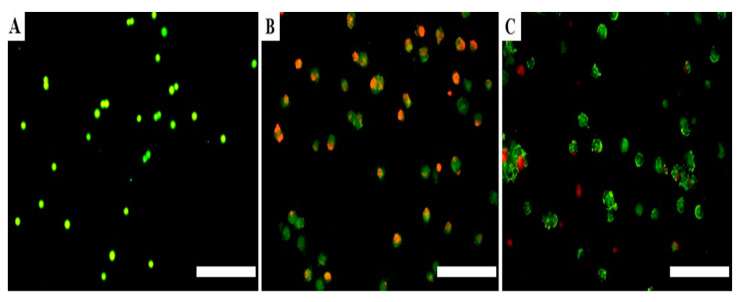
A549 cells with different features of apoptosis as identified with dual staining with AO and PI after 48 h of transfection. MMP8.1.AA and Hs.Cas9.MMP8.1.AB. (**A**) Non-transfected cells, (**B**) Hs.Cas9.MMP8.1.AA (**C**) Hs.Cas9.MMP8.1.AB. Scale 500 µm.

AO/PI double staining of A549 cells by fluorescence microscopy was carried out to determine whether the decrease in the lung adenocarcinoma cells’ ability to migrate and invade was associated to apoptosis. Cells were stained in three independent experiments and then analysed using fluorescence microscopy in order to identify the cell death mode after 48 h of incubation period with treatment. Staining with AO and PI is a technique based on DNA-binding dye, the assay allows the identification of morphological features of four cell stages: Viable cells (stain uniformly green), early apoptotic cells (chromatin condensation with green nucleus stained irregularly), late apoptotic changes (chromatin fragmented or condensed with red stained nucleus) and cells in a necrotic stage (nucleus stained uniformly orange). Based on the morphological description, the non-transfected cells showed a higher number of viable cells and reduced number of apoptotic cells after 48 h of incubation (Figure 4A). Meanwhile, transfection of Hs.Cas9.MMP8.1.AA sgRNA resulted in high number of necrotic cells (Figure 4B). However, a minute number of late apoptotic cells was seen in Hs.Cas9.MMP8.1.AB sgRNA-transfected cells (Figure 4C).

### 3.4. Delivery of Transfection Components Did Not Affect the Cell Viability

**Figure 5:**
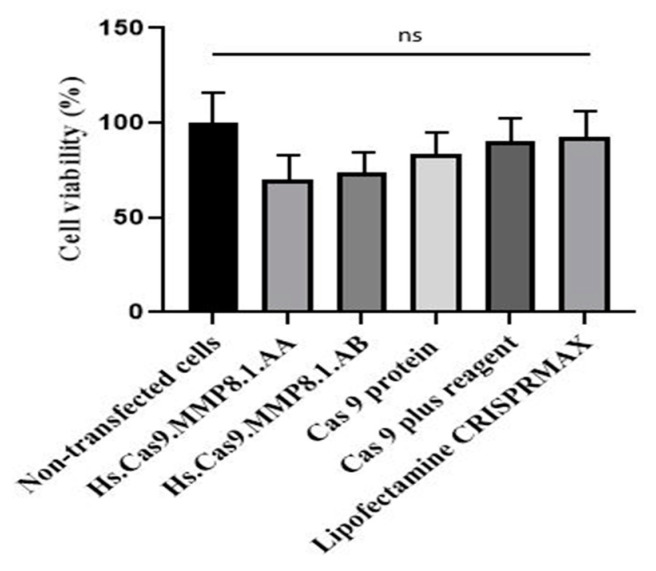
Cytotoxic effect of CRISPR/Cas 9 reaction components on A549 cells lines. Cells were treated with individual reaction components for 48 h and the cell viability was determined by MTT assay. The data were expressed as mean values ± Standard Error of the Mean (S.E.M) of viable cells (*n* = 9).

The cytotoxicity of each transfection component (Hs.Cas9.MMP8.1.AA, Hs.Cas9.MMP8.1.AB, Cas9 protein, Cas 9 plus reagent and Lipofectamine CRISPRMAX) following 48 h of incubation on A549 cell line was examined through MTT assay. This experiment was to confirm that the cell necrosis and apoptosis were specific to the *MMP8* gene targeting following delivery of Hs.Cas9.MMP8.1.AA or Hs.Cas9.MMP8.1.AB sgRNA and its complexes. The results indicated that none of the components demonstrated any significant cellular toxicity on the A549 cells. Non-transfected cells were used as a control group (Figure 5).

### 3.5. Lipofection of sgRNAs and Cas9 Protein Complexes Resulted in the Decreased Expression of MAP-K, Survivin and PI3-K in A549 Cells

**Figure 6:**
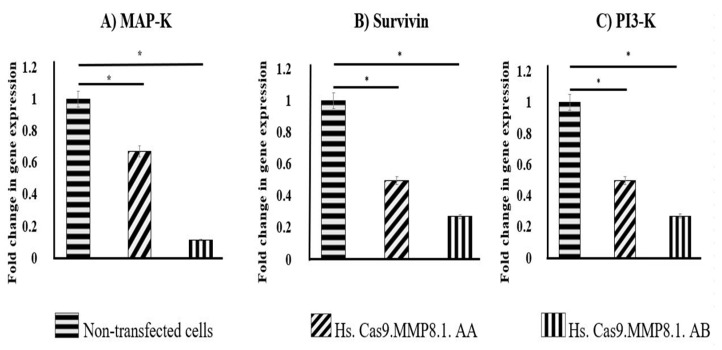
Fold change in gene expression. Reduced gene expression of *MAP-K* (**A**), *survivin* (**B**) and *PI3-K* (**C**) in the groups transfected with Hs.Cas9.MMP8.1.AA and Hs.Cas9.MMP8.1.AB gRNAs compared to non-transfected cells (*p* < 0.05) after performing real time PCR. Data were expressed as mean values ± Standard Error of the mean (S.E.M), (*n* = 3), (* *p* < 0.05).

Real time PCR was performed after transfection to determine the fold changes in the expression of proliferative genes such as *MAP-K, survivin* and *PI3-K* in both transfected and non-transfected groups. Cells transfected with Hs. Cas9.MMP8.1. AA and Hs.Cas9.MMP8.1.AB expressed *MAP-K* at 0.67 (*p* = 0.048) and 0.11-fold (*p* = 0.001) lower level than non-transfected cells (Figure 6A); *survivin* at 0.49 (*p* = 0.047) and 0.26-fold (*p* = 0.038) lower level than non-transfected cells (Figure 6B); *PI3*-*K* at 0.54 (*p* = 0.048) and 0.24-fold (*p* = 0.003) lower level than non-transfected cells respectively (Figure 6C).

## 4. Discussion

Normal lung cells express relatively lower levels of matrix metalloproteinase 8 (*MMP8*), and probably increase its secretion when cells are exposed to a pro-MMP activator during the transformation into carcinoma cells [21]_._ The role of *MMP8* in maintaining normal pulmonary function during inflammation and tumorigenesis is unknown [22]. Despite this, several reports have suggested that *MMP8* might have anti-tumoral properties, by reducing the migratory activity of cancer cells and increasing cell attachment to extracellular matrixes within the tumour [23,24]. Most studies correlate *MMP8* to cancer advancement by determining the mRNA expression in tissue samples, as well as protein analysis in either tissue or blood samples [10]. Therefore, postulating the exact mechanisms on how *MMP8* modulates tumour development is less accurate. Instead, gene targeting experiments are better alternatives to assign functions to a specific gene.

We believe that this is the first study to demonstrate that upon delivery of *MMP8* sgRNAs with CRISPR-Cas protein into the A549 cell line, the cells showed decreased proliferation, as observed by the reduced number and size of forming colonies (Figure 1). Our search in the literature reveals that the expression of integrin, a transmembrane receptor important for cell adhesion, is also regulated by MMP [23]. Integrin is present in the lung epithelial cells and is involved in pulmonary morphogenesis and maintenance of tissue homeostasis [25]. Furthermore, integrins mediate signals that facilitate cell proliferation, differentiation and apoptosis via mitogen-activated protein kinase/survivin (*MAP-K/survivin*) pathway [26]. Here, reduced cancer cell viability and apoptosis are reflected in the results presented in Figure 4 with transfected cells depicting different features of apoptosis, and the data represented in Figure 6 with the reduction in fold changes of *MAP-K* and *survivin* gene expression (Figure 6A,B). It is also possible that the reduction of cell viability was affected by the decrease in *PI3-K* gene expression (Figure 6C). Noteworthy, the function of *PI3-K* signalling pathway has been linked to cancer stem cell maintenance, and might be accountable for failure in chemotherapy [27]. 

*MMP8* has been demonstrated to modulate the chemotactic activity (e.g., CXCL5 and CXCL11) of many cells in physiological conditions [28]. and in metastasis of cancer cells [12]. Our study showed that the lipofection treatment to target on *MMP8* gene reduced the migration potential of A549 cells. Transfection of both sgRNAs recorded significantly lower migratory activities when compared to the non-transfected cells (Figure 3). After 48 h post-transfection, the scratch assay indicated relatively wider wound gaps as well (Figure 2).

In cancer progression, cells lose their adhesion and acquire epithelial to mesenchymal transition (EMT) leading to spread to distant tissues [29,30]. Our results demonstrated that lipofection of sgRNA and Cas9 protein complexes could have induced the knock-down of *MMP8* gene expression, thereby reducing cell-to-cell adhesion, and ultimately decreased cell proliferation. However, instead of progressing to convert to a mesenchymal phenotypic and acquiring enhanced chemotactic activity, inactivation of *MMP8* gene has arrested the migration potential of the A549 cells. Therefore, future studies should examine the possible affected downstream signalling pathways, for instance by performing next-generation sequencing, following *MMP8* gene inactivation.

The limitation of this study is lacking data to show the level of MMP protein secretion or enzymatic activity of *MMP8* following lipofection of the two individual sgRNAs, which specifically target different nucleotide sites. This protein secretion could be affected by lipofection efficiency, which was undetermined in the current study. Prior studies have shown that the presence of *MMP8* variants in different tissues may have anti- or pro-tumoral properties [13]. Therefore, it is possible that these nucleotide sites might have a significant function in modulating cellular events in carcinogenesis. Since *MMP8* could also interact with many cytokines, the mechanisms of how inhibition of *MMP8* may lead to a change of events in inflammatory responses should be investigated as well.

Many synthetic MMP inhibitors, for instances Tanomastat and Rebimastat, have been prescribed to cancer patients in clinical trials. However, the clinical outcomes showed failure in reducing tumoral burden [31]. In this study, we also pointed to the possibility of using CRISPR-Cas9 technology to target specific genes to halt development of tumour that shows different expression profile of MMP, in alternative to using synthetic inhibitors. We have previously used single guide RNA to disrupt the *MMP20* gene expression in lung cancer cells, and successfully showed a reduction in cell proliferation and migration, along with significantly reduced activity of *PI3-K*, and *survivin* signalling pathways [17]. Here, our current study is able to provide alternative gene for efficient cancer cell targeting to achieve similar efficiencies. CRISPR-Cas technology also simultaneously allows correction of a mutated gene upon double-strand breaks by concurrent delivery of normal gene sequences for replacement [32,33,34,35].

In conclusion, the current study demonstrated that matrix metalloproteinase 8 is involved in adenocarcinoma lung cancer progression. When the single guide RNA targeting on *MMP8* gene was transfected with Cas9 protein into A549 cell line, the cells lost its proliferative and migratory activities, in vitro. Further studies are required, in order to delve deeper into the molecular mechanisms and verify anti-tumoral properties in animal models.

## Data Availability

The data presented in this study are available on request from the corresponding author.
